# Seeing on the fly: No need for space-to-time encoding; saccade-generated transients enable fast, parallel representation of space

**DOI:** 10.1167/jov.25.11.4

**Published:** 2025-09-04

**Authors:** Moshe Gur

**Affiliations:** 1 Technion, Haifa, Israel

**Keywords:** saccade, fixational eye movements, image representation, drift, primary visual cortex

## Introduction

In my Perspective (Gur, 2024), I have argued that the visual system can rapidly and efficiently handle a vast amount of information by keeping a parallel, one-to-one, data transfer between the retina and the primary visual cortex (V1). High-resolution vision is enabled by using saccadic eye movements to direct the central retina with its densely packed photoreceptors toward spatial regions of interest (ROIs). Thus, we sample the outside world three to four times per second by repeated cycles of landing and fixating. In V1, the retinal image is represented as a map of responding neurons, so that every image element activates neurons with spatially selective receptive fields (RFs). Once neural activity is evoked, all represented information is available for further processing.

This generally accepted view has been challenged by an increasingly popular school of thought maintaining that space is not represented by the responses of neurons in corresponding locations within the one-to-one parallel map, but by a temporal code generated by the sliding retina during fixational pauses, such that each spatial input element is encoded by the firing pattern of several crossing neurons.

In the Perspective, I pointed out that space-to-time (STT) neural encoding necessarily slows down transmission and representation by deferring processing from the landing saccade evoked response to the end of the fixational pause. I have also presented a host of physiological and behavioral evidence that makes STT encoding untenable.

Rucci et al. (2025; henceforth Rucci et al.) denied my arguments relying on general considerations and an eclectic collection of arguments and rhetoric, rather than on a detailed understanding of visual physiology and psychophysics.

Before responding to the specific comments presented by Rucci et al., it is worthwhile to take a broader view of the rationale for the space-to-time encoding approach.

## Is there a need for a space-to-time encoding?

**If it ain't broke, don't fix it.** Information transfer and representation of the outside world via parallel, space-to-space mechanisms is reliable, consistent, and highly efficient. When neural activity reaches V1, significant spatial processing is immediately performed by spatially selective cells, and further processing can commence without delay. To impose additional processing by STT neural encoding calls for dedicating additional neural resources and significant pauses in upstream processing. How do Rucci et al. explain the need to fix a well-working system?

According to Rucci et al., the fatal fault in the pure spatial “camera-like . . . static” view is in ignoring that time transients are essential for the visual system functionality and that, specifically, the Perspective is wrong in suggesting that the landing saccade creates a “flash-like imprint on the retina.” However, one of the central issues in the Perspective is that the required transients are the powerful time-locked neural volleys generated by the landing saccade. This suggestion is based on solid physiological evidence from various labs. Unlike their unequivocal statement that there is no “shutter-like imprint,” their denial of the role played by landing saccades is vague and avoids the issue. They say, “But saccades are not instantaneous, the stimulus moves over the retina both before and after saccade landing.” How is this relevant? In the Perspective, I argued at some length that upon landing, the abrupt saccadic deceleration creates a flash-like contrast step, but Rucci et al. simply ignore it. It is also telling that they do not say explicitly that the landing saccade does not generate time-locked responses, which they know to be incorrect, but let the readers draw their own conclusions from their statement about stimulus motion.

Before discussing specific issues, we should keep in mind that the entire edifice of the STT encoding is based on the presumed lack of time transients in the pure spatial view. Only by denying that landing saccades provide such strong transients can Rucci et al. justify STT encoding. As stated briefly above and discussed below, both logic and solid evidence show that landing saccades provide the essential time transients.

## The STT theory is all about reshaping the input. How the visual system, V1 neurons in particular, processes these changes is of little interest

In this Letter, V1 organization and single-cell properties embody the way the visual system achieves its exquisite one-to-one, parallel representation of the outside world. This was done because V1 is the only area with high-resolution spatial mapping and feature selectivity, particularly at the foveal representation, that is compatible with human high acuity perception.

The proponents of the STT theory were from its very inception interested in the way fixational eye movements (FEMs) reshape the image to equalize power (whitening) at all relevant spatial frequencies (SFs). How the nervous system encodes the reshaped image was of little interest. Here is what Rucci et al. had to say on this subject (emphasis is mine): “Most fundamentally, spatial information is not lost, it is encoded in the spatiotemporal structure of visual *stimulation*; . . . each movement reformats spatial patterns into spatiotemporal *input* signals. . . . The visual *input* signals reformatted by eye movements discard redundant information in natural scenes *before any neural processing*.”

Thus, the STT theory proposes that saccades and fixational drifts together provide information across a broad range of spatial frequencies, based on the specific way each movement reformats spatial patterns into spatiotemporal *input* signals, “the idea that spatial encoding makes use of visual input transients resulting from eye movements.” This spatiotemporal reformatting occurs before “any neural processing” and is present in the input signals experienced by neurons.

The idea that retinal drift modulates the image for much of the fixational pause before neural processing, as claimed by Rucci et al., sounds impossible since as soon as the retina starts drifting across the image, all relevant neural elements start responding, such that image modulation and neural responses occur simultaneously.

How these input changes are encoded by the visual system, particularly by V1 cells, does not seem relevant to the STT theorists. The occasional reference to the working of the visual system is to acknowledge that photoreceptors are needed to process visual inputs: “This means that the temporal structure of activation of receptors is as important as their spatial locations” (Rucci et al.) or that retinal ganglion cells (RGCs) are involved: “these dynamics generate transient signals that are well-matched to the sensitivity of retinal ganglion cells” (Mostofi et al., 2020). How visual information is handled by V1 neurons is ignored, with the telling exception of using a model simple V1 cell with a >2° RF lacking side inhibition (Boi, Poletti, Victor, & Rucci, 2017).

However, this lack of interest in neural processing may have led to the rather absurd situation where, despite intensive work spanning more than two decades on how drift shapes visual input, there is a total indifference to the presumed neural code that must be generated by the visual system to take advantage of the modulated image. The only neural model on this general issue was proposed by Ahissar and Arieli (2012), but it has nothing to do with the whitening effect but instead with the puzzle of seeing high SFs despite the expected smearing by the moving eye. Furthermore, Ahissar and Arieli's model was not quite realistic in ignoring saccades, using long repeating cyclical drifting motion and assuming no variability of any kind.

This no-model practice forced me, both in the Perspective and in the current effort, to engage in shadowboxing when trying to show the implausibility of neural implementation of the STT encoding. For example, even in suggesting a rudimentary mechanism, the proponents of the STT theory would have needed to specify several parameters: the number V1 cells that must be activated by the drifting retina, the required drift amplitude per pause, how to overcome large response variabilities, and more. Thus, Rucci et al. have not tried to counter my arguments by presenting even an outline of a model but opted instead to try to undermine the validity of my arguments.

## The STT theory necessarily discards the benefits of the pure spatial scheme

It is rather perplexing that to reap the presumed benefits of image whitening, Rucci et al. discard the built-in advantages of the space-to-space mechanisms while accepting the limitations imposed by STT encoding, resulting in a visual system that operates slowly, inefficiently, and unreliably. Does image whitening confer such advantages to justify these shortcomings? In fact, it is easy to show that the presumed benefits of STT encoding are achieved by the very structure of the primate visual cortex.

Rucci et al. also state that “unlike the camera-like model, which unrealistically requires all spatial information to be simultaneously transmitted to the cortex through the limited-capacity channel of the optic nerve.” Obviously, in their all-out effort to discredit the space-to-space view, they portray its proponents as being unaware that the retina is strongly anisotropic and that the main function of the saccade/drift cycle is to bring the high-capacity foveola to sample a small part of the visual field.

As Rucci et al. tell us, for many years, the dominant view regarding visual information processing was the pure spatial one. This view holds that there is a one-to-one relation between spatial elements and single neurons in the visual system. In V1, for example, the outside world is projected onto a two-dimensional (2D) map such that properties of elements in the world are represented by the identity of responding cells with appropriate RF characteristics. According to my version of the spatial hypothesis, the parallel one-to-one cortical spatial representation is activated by V1 cells that respond to the abrupt luminance (and color) change generated by the landing saccade.

Since information is represented by the identity of responding cells, such an arrangement has several built-in advantages:

**Speed.** Representation and consequent processing may be initiated by the very first spikes generated 30 to 50 ms from saccade landing (Resulaj, Ruediger, Olsen, & Scanziani, 2018) and peak responses being reached 10 to 20 ms later.

**Efficiency.** At the limit, each element's location and size may be represented by a single cell with an appropriate RF that is tuned to a particular input feature.

**Immunity to response variability.** Since information is represented by the identity of responding cells, the timing or exact nature of the response is not important. Thus, the large variability in response strength or timing (see below) is not a problem.

Rucci et al. suggest that the space-to-space notion should be abandoned since STT encoding confers significant benefits on the visual system—most notably, natural image whitening, and hail the “the exquisite efficiency of the mechanisms attuned to the spatiotemporal signals generated by the various classes of eye movements.” Coding space by time, however, means that the advantages of the space-to-space scheme listed above are lost. Here is why.

**Speed.** It takes time for the retina to slide over the image during fixational pauses to generate an STT code. Rucci et al. insist that I was wrong in assigning a short effective interval and that encoding may last the whole ∼250-ms pause. Kuang, Poletti, Victor, and Rucci (2012) state that a 512-ms interval would be insufficient to enhance SFs at temporal frequencies <4 Hz. Since we perceive space as such rather than a collection of time sequences, additional time is needed for decoding the time code back into space (see below). So rather than enable processing within 30 to 50 ms from saccade landing, STT encoding would delay processing by hundreds of milliseconds.

In other words, when a pure spatial representation is replaced at every location by a sequence of pulses in several drifting cells, the strict space-to-space, one-to-one, parallel representation is temporarily gone.

**Efficiency.** To encode a feature, the retinal image must slide across, say, four cells (Ahissar & Arieli, 2012). So, if a feature is represented by N cells in the space-to-space approach, it will take 4N cells in the STT view. In addition, Rucci et al. suggest that to overcome response variability, population averaging is needed. Although the population size is not specified, let's assume 25 cells as a very conservative estimate. So rather than the N cells required in the space-to-space approach, the STT would call for 4 × 25N, a 100-fold increase—a totally unrealistic design.

**Variability.** While variability in response amplitude and onset timing may, in theory, be overcome by dedicated populations, it is hard to see how variability in participating neurons, resulting from the erratic nature of the drift or from the retina landing at different locations, can be compensated by population coding.

## Suppression of low-to-mid SFs is achieved by primate V1 neurons. There is no need for input whitening

The raison d’être of the STT approach is that image modulation by FEMs is beneficial to visual processing by equalizing spatial frequencies (whitening) and discarding redundant information during the viewing of natural scenes. For this transform in SFs to be the dominant factor in shaping visual responses, neural cells are treated as mostly passive elements that follow luminance modulations at each frequency band. Thus, when discussing visual processing, photoreceptors or RGCs are usually referred to. When a V1 cell is used in a simulation (Boi et al., 2017), it is carefully chosen, a simple cell with a very large (>2°) RF with no surround inhibition, so that it is not inhibited by very low SF stimuli. However, most primates’ V1 cells are complex with very small RFs at the foveal representation (cf. Gur & Snodderly, 2008; Hubel & Wiesel, 1968, Hubel & Wiesel, 1974). Since this practice ignores the physiology of cortical neurons, a short reminder is called for.

Photoreceptor responses are a function only of absorbed photons since, devoid of surround inhibition, they are not affected by the spatial properties of the stimulus, as long as it is equal to or larger than their RFs. RGCs with their center/surround RFs respond optimally to a center-size stimulus but will also respond strongly to any wide-field one. A similar organization is found in lateral geniculate cells, albeit with stronger surround suppression. A dramatic change in RF properties takes place in the primary visual cortex, where cells are very selective to the spatial attributes of the stimulus and, most importantly, are strongly inhibited by stimuli extending beyond their RFs. Here is what David Hubel (1988) had to say on this issue: “Whereas many geniculate cells respond to diffuse white light, even if weakly, cortical cells, even those first-stage cells that resemble geniculate cells, give virtually no responses.” Hubel and Wiesel (1968) show several examples of strong inhibition by stimuli extending into the cell's inhibitory surrounds (cf. Figures 3 & 4, ibid).

**Figure 1.**
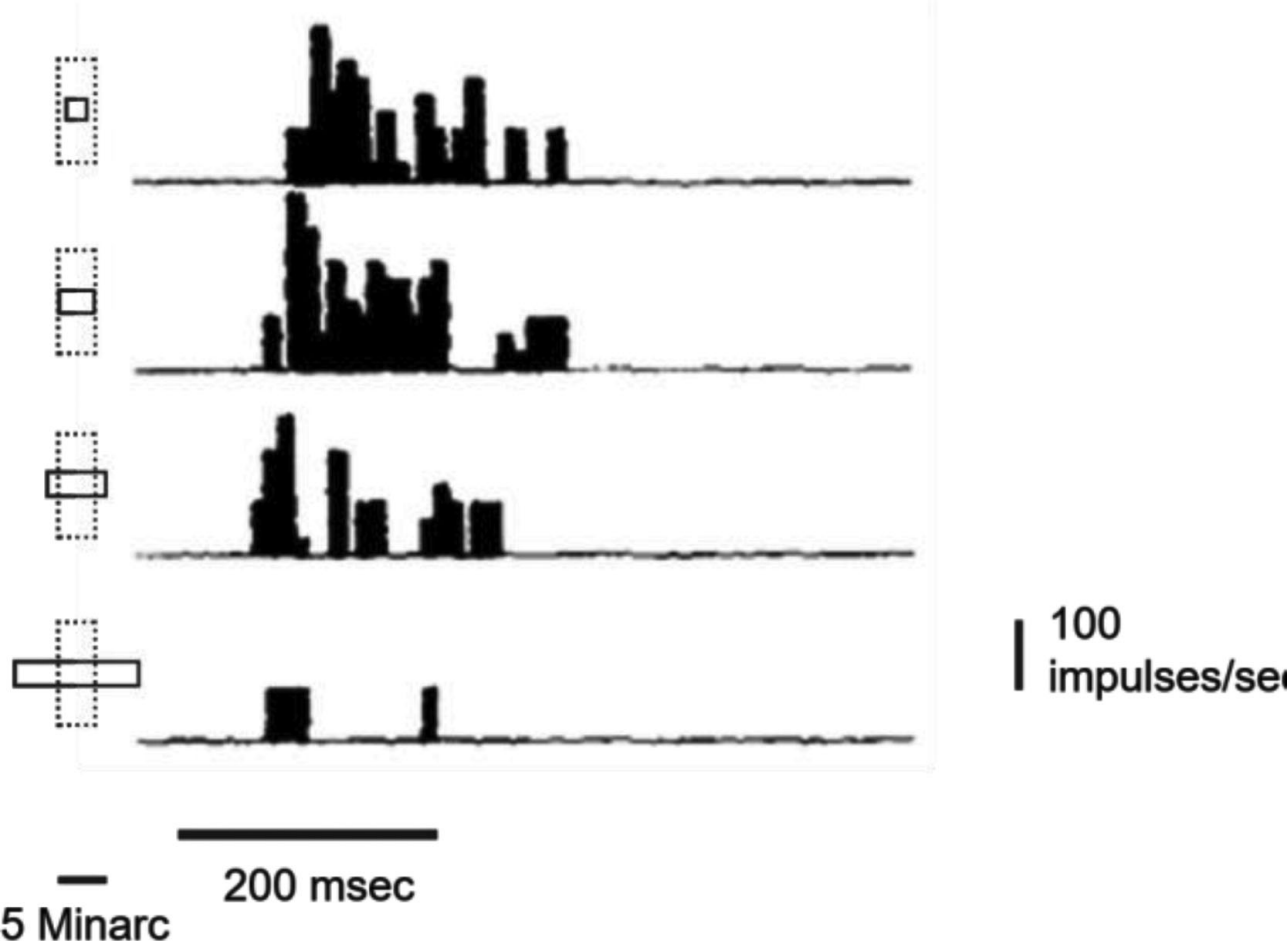
Effects of stimulus size on responses of a vertically oriented RF in V1 of a behaving monkey. From Gur and Snodderly (1987, Figure 4).

Figure 1 shows a striking example of suppression by stimuli larger than the 4′ RF where a bar, although orthogonal to the cell's preferred orientation, is very effective as long as it stays within the RF boundaries but is very suppressive when extending, even partially, into the inhibitory surround. It is likely that the large pyramidal cells in the monkey's V1 layer 3 feature the smallest, most finely tuned RFs in the entire visual cortex (Gur & Snodderly, 2008).

We can thus see that feature selectivity in V1 cells is determined by the spatial organization of their inputs, but it is strongly controlled by surround inhibition so that any deviation from the RF boundaries (length, width, orientation) eliminates responsivity. What decides a V1 cell response is how well the stimulus shape matches its RF. If an image element is larger than the RF, increasing or decreasing its luminance will not matter.

In V1 output layers, RF widths within the foveal representation are quite small, ∼4–8′ (cf. Gur & Snodderly, 1987, Gur & Snodderly, 2008), and are even smaller within the foveolar representation, presumably down to a 1–2′ width (Gur, unpublished observations). Thus, low to mid-SF suppression is achieved very effectively by the very spatial organization of V1 RFs independent of any image reformatting.

**Figure 2.**
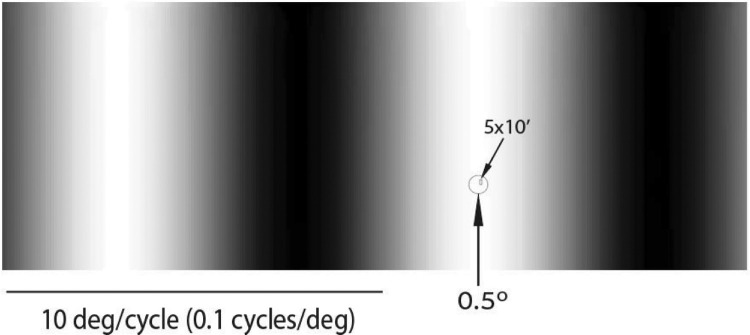
Contrasting a low spatial frequency (0.1 cycles/deg) grating with the foveola (0.5 deg) and with a 5 × 10′ foveolar receptive field.

**Figure 3.**
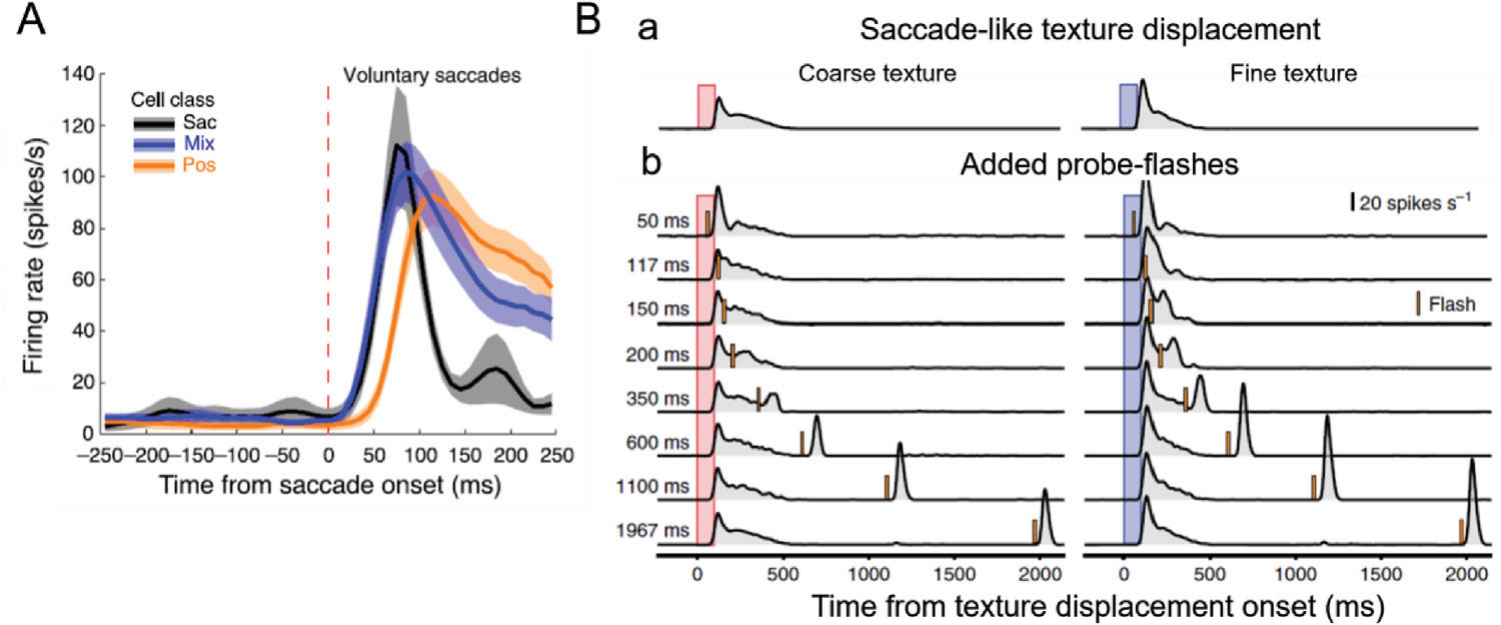
(**A**) Averaged single-cell responses to saccade landing in the monkey V1. Cells were classified according to their responses to a stimulus landed on and staying within the landing RF: “Sac” cells responded only to the landing saccade; “Mix” cells responded to the landing saccade and, occasionally, during drift; and “Pos” cells responded strongly to the landing saccade and consistently to drift. Note the strong decay following responses to the saccade landing. From Kagan et al. (2008, Figure 3A). (**B**) Decrease of mouse RGC responsivity following a saccade-like shift across coarse (pink bars) and fine (blue bars) textures. (a) RGCs’ averaged responses to saccade-like shifts of coarse and fine textures. (b) Responses to shifts followed by pulses delivered at different intervals after the shifts. It takes ∼600 ms after the saccade-like shift for the retina to recover full sensitivity. Data from Idrees et al. (2020) show strong transient responses to both saccade-like shifts and flashes on both coarse and fine textures. That responses to flashes are more transient than responses to shifts may be attributed to the difference between averaging the more variable responses to mechanical shifts versus tighter responses to electronically generated flashes. From Idrees et al. (2020, Figure 3).

Here is an illustration showing that a 0.1 cycles/deg (10 deg/cycle) grating, which, according to Mostofi et al. (2020; Figures 2–4; see also Rucci et al., Figure 2C), is well within the range of low SFs preferably suppressed in the saccade-reformatted image, is practically a diffuse blank field when landing on a foveolar RF. Obviously, a 5 × 10′ RF will be strongly inhibited, far beyond any possible effects of a saccade-induced suppression.

To summarize, while the pure spatial hypothesis is compatible with a fast, efficient, and reliable transmission and representation, the STT approach results in a slow, highly inefficient visual processing. Rucci et al. simply ignore obvious shortcomings to preach the importance and necessity of the STT theory, not by concentrating on physiological and psychophysical evidence but by characterizing the pure spatial view as being an unsophisticated, old-fashioned one, “a reductionist approach . . . static . . . a traditional camera-like view . . . naive introspection.”

## A response to the critique of Rucci et al.

In the Perspective, I have listed a few features of the saccade/fixation cycle that make the STT theory untenable. Rucci et al. have responded with an interesting mix of relevant and irrelevant counterarguments. Here are the main issues.

### The effects of landing saccades

As noted above, the only real argument against the pure spatial view is the lack of a “shutter-like” signal, without which the visual system remains static and unfunctional. To the data-derived argument in the Perspective that such a signal is provided by the responses to the landing saccades, Rucci et al. respond not by denying these responses but by saying that “saccades are not instantaneous, the stimulus moves over the retina both before and after saccade landing.” It is thus worthwhile to look closely at the evidence for saccade-generated responses.

In the Perspective, I have presented data from three studies showing responses to both flashes and saccade landings in the monkey V1 (Gawne & Martin, 2002; Kagan, Gur, & Snodderly, 2008; Ruiz & Paradiso, 2012). All three studies show that the response to saccade landing is high and stays higher than baseline activity. Furthermore, data from Idrees, Baumann, Franke, Münch, and Hafed (2020) show that a few hundred milliseconds after the response to a saccade-like shift, the threshold for responses generated by other stimuli, flashes, is high. This elegant study reflects the well-known property that when the resting potential is above threshold, the cell excitability is reduced. Evidence consistent with single-cell recordings was presented by Slovin, Arieli, Hildesheim, and Grinvald (2002), who used voltage-sensitive dye imaging to show strong transient responses to landing saccades in the primate V1, and by Kaunitz et al. (2014), a human VEP study where saccades elicited strong transient responses.

Thus, physiological evidence clearly shows that the landing saccade generates in V1 cells powerful, fast-rising, phase-locked volleys. This response starts ∼50 ms after saccade onset, reaches its peak 10–20 ms later, and, most importantly, stays above baseline for practically the rest of the fixational pause (cf. Rucci et al., Figures 2A, 2B, and Figure 3 in this Letter). When the fovea lands on a new ROI, each V1 RF that is stimulated by the appropriate image feature responds robustly, and this interaction takes place within a single RF border (see below)—as suggested by the space-to-space approach. Although responses to the landing saccade dominate the entire drift period, since a given element stays within the landing RF, any additional responses that may be generated by drift merely add strength to the responding cell. In other words, the strong, short-latency responses initiated by the landing saccades implement the one-to-one spatial representation by V1 neurons.

### Rucci et al.’s counterarguments

**Saccades take time** **and** **affect low SFs.** Responding to my data-based description of landing saccades as generating powerful neural volleys, Rucci et al. ignore all physiological evidence in favor of input modeling that is based on far-fetched assumptions. They claim that *saccades are not instantaneous* *.* *.* *.* that they *.* *.* *.* *last tens of milliseconds* . . . and that they extend *. . .* *sensitivity to lower spatial frequencies than drift.* Their most telling statement is, “Mostofi et al. (2020) does not deal with perceptual or neural responses at all. It is a power spectrum analysis of the visual input to the retina.”

They also present a broad view of shaping the entire SF input spectrum by the combined effects of saccades and drifts: “Vision relies primarily on saccade transients at low spatial frequencies and on the integration between saccade-induced and drift-induced modulations, leading to coarse-to-fine dynamics.”

As noted above, Rucci et al. are perfectly content to show how the input may be reformatted by FEMs as if the visual system is made of arrays of passive sensors that follow the amount of input energy contained within each spatial frequency band. Even after I cited studies that clearly show that saccades generate short, powerful volleys, they turn a blind eye and keep arguing that only input changes matter. For example, while Boi et al. (2017) repeatedly state that the combined effects of saccades and drift in reshaping the image lead to “coarse-to-fine” perception dynamics, they do not bother to specify the periods of the two stages. When I noted this lack of specification, Rucci et al. assure us that “Figure 3E in Boi et al. shows a window of saccade influence of approximately 50 ms.” Well, maybe it shows, but certainly does not specify.

The physiological consequences of the landing saccade are noted only when Rucci et al. try to disparage the Perspective by saying that, for example, the schematic Figure 2 does not depict V1 responses accurately enough, that saccades differ from flashes, or that there are three classes of cells in publications that I coauthored.

In trying to depict a smooth, continuous transition (“integration”) between landing saccades and drifts, Rucci et al. equate a very fast saccadic sweep over a wide spatial expanse with an extremely slow drift over a very limited area. In boldly claiming that the only difference between the two types of movements is their velocity, Rucci et al. manage to create a continuum. To state the obvious, there are no similarities between the physiological effects of saccades and drift. At saccade onset, all responding cells stop firing (cf. Snodderly, Kagan, & Gur, 2001), and importantly, the >200°/s retinal motion means that no visual cell can respond to any crossed spatial element (cf. Priebe, Lisberger, & Movshon, 2006, for a 64°/s upper limit for V1 cells’ responses). The fast sweep across space does not elicit any responses in visual neurons, while, during fixational pauses, the retina drifts slowly across potentially responsive cells. The two epochs, the large amplitude, very high velocity saccade, and the extremely low-amplitude, slow-moving drift are so different in their physical characteristics and potential neural effects that the attempt to link them as one continuous phenomenon is misleading.

What is glaringly missing, although clearly addressed in the Perspective, is the event that generates the initial responses—the strong deceleration that stops the fast-moving eye in a few milliseconds. Only when the fovea lands on a selected ROI can the silent neurons experience and respond to the 1- to 2-ms sudden change in luminance. This flash-like stimulation leads to immediate, powerful responses. So, unlike the assertion by Rucci et al., saccade landings do provide a clear, effective signal that is synchronized with our exploration of space. Such a signal prevents fading and smearing and carries the information necessary for the space-to-space, one-to-one parallel neural representation.

## There is little time for drift-generated spikes

### How long is a useful fixational pause?

In the Perspective, I have used an estimate of the saccade/fixations cycle duration, 250 ms, which reflects the average interval found in several studies. However, looking at the standard deviation in those studies, we realize that many fixations are much shorter: 248 ± 54 ms (Kuang et al., 2012), 264 ± 78 ms (Boi et al., 2017), and, most impressively, for the best-performing subject in Gersch, Kowler, Schnitzer, and Dosher (2008), fixation durations were considerably shorter—197 ± 57 ms. So, 16% of all successful fixations in those studies lasted, at most, 194, 186, and 140 ms, respectively.

In many, if not most, fixations, the image is processed and analyzed, and objects are recognized before the next target is selected and planning for the next saccade begins. Searching for a face in a crowd is a case in point. It is estimated that planning for the next saccade starts ∼100 ms before the next saccade is executed (Rolf & Carassco, 2012).

In V1, it takes 30–50 ms from saccade landing until the first spikes are generated. Rucci et al. estimate that the landing saccade dominates the saccadic pause for the first 50 ms. So, if we consider that for many successful fixations, the saccadic pause is ≤200 ms, that preparation time lasts ∼100 ms, and that responses to the landing saccade dominate the first 50 ms of the response, we can conservatively estimate that the effective drift interval lasts ∼50 ms.

### How much does the eye drift during a pause?

Obviously, there is quite a large range of drift amplitudes during a saccadic pause. However, a mechanism, such as STT encoding, purporting to explain information processing carried out by the drifting eye, should accommodate the total range of drift amplitudes. Many studies show steady fixations during the normal saccade/drift cycle. In Rucci and Desbordes (2003), the mean drift amplitude for three subjects was 4.5′ during a 500-ms fixation and 7.7′ during a 2,000-ms fixation; in Ratnam, Domdei, Harmening, and Roorda (2017, Figure 2), four subjects’ drift amplitude during 750-ms fixation was ∼7′. Skavenski, Hansen, Steinman, and Winterson (1979, figure 18) showed that even when fixating without a bite-bar, drift amplitude during a 1-s period is <10′ (my estimate). Examples of individual fixations in (Rucci, Ahissar, & Burr, 2018), Poletti (2023), and Rucci et al. (Figures 1A–C) show drift amplitudes <10′ during fixation periods of ∼1,000 ms. In contrast, a fairly large drift amplitude was demonstrated in Kaung et al. (2012), where the average amplitude for a 250-ms fixation was ∼11′ (44′/s).

Given the above analysis, it would be entirely correct to ask whether any STT scheme can generate a code during a 50-ms interval with a drift extending 10′/s.

The strong surround suppression found in V1 RFs (see Figure 1) means that effective stimuli must match the RFs’ spatial selectivity (width, length, orientation). When an input element lands on a matching RF, a robust, transient volley is generated. If drift moves even part of the stimulus beyond the RF's boundaries, the cell's response would quickly diminish due to surround suppression. Note that the same stimulus cannot elicit responses from the abutting RF until it is almost completely within its boundaries, away from the suppressive surround. If the stimulus is smaller than the RF, its postlanding drift may generate some additional spikes. To consider an extreme case, during a 0.5′/50-ms drift, a 1′ (30 cycles/deg) stimulus, landing within a very small 1.5′ RF, would barely cross the RF boundaries, let alone stimulate the next RF. So, during the 50-ms effective drift window, all input elements elicit robust responses only from neurons that were activated during landing.

### Counterarguments

“First, Gur missed that the stimulus was not presented at the very center of gaze, where cones are the smallest.” I've shown (above) that during an “effective drift” interval, the retina can barely cross even 1′ target with a 1.5′ RF. “Second, this statement enables estimation of the span of motion, not the speed: ocular drift resembles Brownian motion, and to cover this average span, instantaneous speeds must be much faster.” I concur completely, but the issue is not the level of instantaneous speed but the drift's span, that is, how many encoding cells may cross an object? Moreover, if drift is confined to a single RF, a fast movement simply increases the response of that cell—in accordance with the space-to-space approach.

**Fixational pauses can be quite long.** Rucci et al. maintain that there is a broad range of fixational pauses. So presumably, why do I choose to concentrate on the shorter ones? As pointed above, the STT model should function at all successful pauses, not just at the longest ones.

Rucci et al. add two counterarguments that have nothing to do with the issues at hand. In the first, we are told that in some experiments (Kagan et al., 2008), monkeys were trained to fixate for long and variable drift periods. True. In fact, in all our experiments with behaving monkeys, the animals were trained to hold fixation for 5 s. I fail to see how this information pertains to the present discussion. The second argument holds that if pauses in the normal saccade/drift cycle are too short for an STT code, there are other kinds of pauses, specifically in task-dependent fixations, that are much longer. The Perspective, however, deals specifically with the normal fixation/drift cycle, so why bring up an unrelated issue? Furthermore, it makes no sense to depict task-controlled long drifts as generating an STT time code. Such a proposal seems to defeat the suggested mechanism of guiding drifts to enhance acuity, rather than effecting immediate recognition and localization of a target via saccade-generated volleys and then using long drifts to enhance acuity; the drift period is utilized just to identify a target.

**Jitter is effective in generating spikes.** “Moreover, the many illusions of apparent jittery motion at fixation would obviously not be perceivable if the visual system were not sensitive to the motion signals caused by fixational eye movements. There are several papers in the literature showing high precision in neuronal firing once fixational eye movements are included in the analysis. Segal et al. (2015). . . Greschner, Bongard, Rujan and Ammermuller (2002). . .Wu et al. (2024) [address] exactly the question of encoding precision in the primate retina, concluding that fixational eye movements enhance the precision of visual information.”

Rucci et al.’s response is nothing but a misdirection. They are fully aware that the Perspective, as well as many of the STT theory proponents’ publications, are about the normal saccade/fixation cycle, and yet they present studies showing that drift (eye jitter) per se, without preceding saccades, can produce spikes. No surprises here, but irrelevant to my argument that following the landing saccade, there are either no drift-related spikes or, if there are any, they add to the initial response.

**Decoding.** In response to my mentioning, in passing, that decoding the STT code generated during fixational pauses would require additional processing time, Rucci et al. unhesitatingly state that “there is no need for explicitly decoding spatial information; it is not lost.” Since space is not perceived as an aggregate of temporal codes but as a parallel one-to-one, 2D or three-dimensional spatial entity, the need for decoding is obvious. Indeed, Rucci et al., in disregarding their own statement, say that “spatial information can be efficiently decoded.” Furthermore, Rucci and Victor (2015) ask, “How is information from oculomotor transients decoded?” and Boi et al. (2017) state that “this view implies important mechanisms for decoding visual signal beyond those commonly postulated.” Ahissar and Arieli (2012) present detailed, specific decoding mechanisms, whereas Rucci et al. (2018) ask, “Do saccades establish a clock for visual decoding?”

**It is impossible to distinguish between responses to saccades and to drift.** Rucci et al. claim that once a saccade lands the RF onto a stimulus, it is impossible to distinguish the response to the saccade, which they inexplicably describe as “mere presence of the stimulus within the receptive field,” from responses to drift. They rely on two publications that I coauthored (Kagan et al., 2008; Snodderly et al., 2001). What they fail to understand is that these publications clearly show not only strong time-locked responses to both fixational and landing saccades but that the drifting eye generated additional spikes only as long as the stimulus stayed within the boundaries of the RF. These spikes merely add to the responsivity of the cell that landed on a specific target, in accordance with the pure spatial, point-to-point representation. As noted in the Perspective, the number of drift-generated spikes is small relative to that produced by the landing saccade (cf. Figure 3) and may be useful only during part of the drift period.

## When the retina drifts across space, neural responses are, necessarily, highly variable

In the Perspective, I have noted that several drift-related features make a consistent, reliable STT code utterly impossible. Drift trajectory, span, and velocity vary within a single fixation; the eye lands on different spatial locations even when repeatedly fixating the same target; response amplitude and latencies are highly variable, and, as indicated by Rucci et al., the stimulus position varies relative to the RF.

Note that our ability to accurately perceive spatial elements does not vary. These subtly different three letters, ***a*** *a* a, are consistently perceived and do not vary whether fixated twice or a hundred times. Such consistency cannot be explained by any mechanism that is bound to produce variable results. This conclusion is not only logical but also self-explanatory, yet Rucci et al. nonchalantly write, “Again, this assertion is made without any accompanying logical explanation as to why variability in eye movements should make use of temporal information impossible.” Note that while I argued that variability in neural responses presents a problem, Rucci et al. talk about “variability in eye movements.”

As discussed above, Rucci et al. scrupulously avoid providing even a hint of how an STT code may be produced. This practice enables them to present a mixed bag of counterarguments that are quite irrelevant*.*

### Counterarguments

**It's all about the input*.*** “Most fundamentally, spatial information is not lost, it is encoded in the spatiotemporal structure of visual stimulation.” While I presented insurmountable difficulties in generating a consistent STT code by the responding visual system, the first “fundamental” counterargument is that the input is not affected. How is this relevant?

**RGCs respond to drift in useful ways.** “Spatial information is also present in the instantaneous pair-wise correlation between responses . . . as neurons will tend to be synchronized when they simultaneously cross a contour (Greschner et al., 2002; Segal et al., 2015). . . . The recent study of Wu et al. (2024) addresses exactly the question of encoding precision in the primate retina, concluding that fixational eye movements enhance the precision of visual information.” As discussed above, Rucci et al. cite studies that show responses to jitter that was not preceded by saccades. Such studies have nothing to do with the normal saccade/fixation cycle. Why bring them up?

**Long task-dependent drift.** “Moreover, drifts appear to be both controlled in a task-dependent manner and monitored via extra-retinal signals that contribute to fine spatial judgments.” Again, they cite studies of task-controlled drifts that have nothing to do with the normal saccade/fixation cycle.

**Population response.** “Population response latencies are likely to have a much smaller variability than single-neuron responses.” Do Rucci et al. seriously consider that to overcome just one source of variability, single neurons must be replaced by a neuronal population? As they carefully avoid describing any STT model, such suggestions are meaningless.

**Direction reversals*.*** “And there is also evidence that direction reversals increase drift-based temporal information (Rivkind, Ram, Assa, Kreiserman, & Ahissar, 2021; Gruber, Ullman, & Ahissar, 2021).” The theoretical study by Rivkind et al. (2021) found that curved trajectories improve visual acuity compared to straight ones; Gruber et al. (2021) used 3-s fixations to conclude that drifts are included in a motor-sensory loop. Both studies have nothing to do with the normal saccade/fixation cycle*.*

**Timing is better** **than** **that rate.** “Under anesthetized/paralyzed conditions, response timing changes of only 10 ms can be informative about contrast.” How does finding that changes in response timing are more informative than response rate relevant to the effects of response variability on time coding?

**Retinal position relative to the image varies.** “Gur's claim neglects the fact that, to reach reliable conclusions about variability, one needs to accurately know where the stimulus is relative to the receptive field.” I concur and thank Rucci et al. for pointing out an important source of response variability that I have added (see above) to the possible sources mentioned in the Perspective.

It is clear that Rucci et al. have nothing of substance to say about the many sources of response variability that render an STT code impossible.

## Flashes mimic landing saccades in onset dynamics and neuronal responses

In the Perspective, I pointed out that in the 1–2 ms that takes a saccade to land on a target, the resulting luminance change is flash-like. The similarity between the responses to flashes and landing saccades found in several single-cell studies (see Figure 3; Rucci et al., Figures 2A, B) and in the many behavioral studies that show excellent perception of a wide range of flashed objects is consistent and supportive of the view that the neural volleys generated by the landing saccades provide the necessary information for the one-to-one exquisite representation of space in V1.

### Counterarguments

**Saccades deliver different input than flashes.** “Critically, saccades and flashes are very different in terms of the visual signals they deliver to the retina . . . brief flashes are such powerful stimuli because they generate transients exceptionally rich in spatial information. Unlike flashes, the luminance transients from eye movements produce a major reformatting of spatial information. . . . This spatiotemporal reformatting occurs before any neural processing and is present in the input signals experienced by neurons.” Rucci et al., again, insist that since, according to their analysis, saccades reformat the input while flashes “generate transients exceptionally rich in spatial information,” the latter are unnatural stimuli that cannot provide any useful information on the normal behavior of the visual system. However, that extremely short flashes (<50 µs; cf. Greene & Wisani, 2015), as well as much wider luminance steps, with 1- to 3-ms rise times (see examples in Gur, 2024), are faithfully perceived, shows that perception does not depend on the “transients exceptionally rich in spatial information” that characterize extremely short pulses. CRT or LCD monitors’ generated pulses are very much like the 1- to 2-ms luminance change effected by saccade landing. This similarity is manifested in very similar physiological responses. Rucci et al. try, at some length, to show that responses to flashes and saccades are not identical, but there is no reason why they should be; electronically generated flashes are bound to produce responses that vary little in latency and amplitude, while saccades, being biological processes, are more variable, leading to more variable responses. When averaging, responses to saccades would result in a smeared average (cf. Rucci et al., Figure 2B). When such variability is overcome by averaging according to peak responses, differences between the two response types practically disappear (see Kagan et al., 2008, figure 6).

**Responses to saccades and flashes are similar because “stimuli were presented over blank fields** **. . .** which should contribute to make retinal stimulation in the two conditions more similar to each other.” In the Perspective, I have cited a study by Thunell and Thorpe (2019) where objects could be recognized when presented in a very fast, 8-ms sequence, resulting in images that were not presented on blank fields but, practically, on previous ones. Idrees et al. (2020) showed similar responses to saccade-like shifts and flashes on textured fields (Figure 3B). The two attached demos show excellent visibility where no blank fields exist. The drifting demo shows a drifting grating that presents every 16.7 ms (for a 60 Hz LCD screen) a new, slightly phase-shifted grating without any intervening blanks. It is possible to reduce the grating such that it extends across 5% of the screen with a ∼0.8-ms rise time or to enlarge it to occupy 60% of the screen with a 10-ms rise time without affecting perception. Similar considerations apply to the rotating demo where the grating rotates by 5° every 16.7 ms.

Our excellent perception of flashed information is not due to artificially generated time-transients but a direct result of the way our retinas are stimulated: In both saccade landings and flashes, there is a fairly abrupt interaction between the image and retinal cells, caused by the sudden landing of the eye on a new object (natural viewing) or by the fast rise time of the screen-generated image. Thus, when a saccade lands the retina on an object, the result is very much flash-like.

**Saccades differ from flashes in physiological responses*.*** “Kagan et al. actually showed important differences in the effects of the two types of stimulation (Figure 2A). While physiological data indicate that both landing saccades and stabilized contrast steps can yield similar magnitudes at their peak responses (Figure 6A in Kagan et al.), the dynamics of neural activity differ considerably: even transient neurons exhibit a shifted and more sustained response following saccades, when the stimulus on the retina moves normally because of fixational drift.”

Figure 6B in Kagan et al. actually shows similar dynamics in latencies and decay levels when responses to all saccades are considered. So, the very publication that Rucci et al. cite shows that physiological responses to saccades and flashes are very similar in magnitude and dynamics. It is not surprising that for a subpopulation, transient cells, there are minor differences. But it is puzzling that Rucci et al. use a subpopulation that does not respond to drift in support of the presumed benefits of the drifting eye.

## There is no evidence in support of the space-to-time theory

After reading Rucci et al.’s arguments reassuring us that indeed there is ample evidence supporting the validity of the STT theory, it is clear that there is not a single study that shows that during a normal saccade/fixation cycle, the drifting eye provides any perceptual benefits.

In the Perspective, I have noted that all empirical data presented by the proponents of the STT approach are fundamentally flawed since performance was tested for periods that were much longer than the ∼200-ms normal drift interval and since stimuli were displayed as a train of short pulses, which prevented any effective image/drift interactions.

How did Rucci et al. respond?

**“There is a large and growing body of evidence**, ranging from human psychophysics to neurophysiology, supporting the notion that oculomotor transients provide useful spatial information. The interested reader is referred to Intoy et al. (2024), Yang, Intoy, and Rucci (2024) and Wu et al. (2024) for the most recent experimental validations of theoretical predictions.”

Rucci et al. cite three studies that are clearly irrelevant since they are not about normal saccade/fixation behavior; in both Intoy et al. (2024) and Wu et al. (2024), there were no saccades, while Yang et al. (2024) studied the effects of blinks. Furthermore, as is the case in all studies related to the presumed advantages of the drifting eye, tested intervals were much longer than in normal fixations. Subjects in Intoy et al. and Yang et al. were tested after fixating for more than 1,000 ms. Other works, presumably part of the “large and growing body of evidence,” employed the same practice; Rucci and Desbordes (2003) used 500- or 2,000-ms intervals; Rucci, Iovin, Poletti, and Santini (2007), 1,000 ms; Segal et al. (2015), 2,000 ms; and Boi et al. (2017), 800 ms. We see that Rucci et al. do not challenge my claim that no useful information is generated by drifts during the ∼250-ms saccade/fixation cycle. Whether drifts can be useful under other circumstances is not the issue here.

I have also argued that when presenting stimuli on a CRT display, the retina is not drifting across the image but rather is experiencing a series of short pulses, in both stabilized and nonstabilized conditions. Since showing that performance deteriorates under stabilized, nondrifting stimuli is how the STT proponents demonstrate the advantages of the drifting eye, Rucci et al. responded by saying, “At a more conceptual level, Gur's intuitive assumption that the stimulus is “frozen” on the retina and cannot be reformatted by eye movements when displayed via a train of brief flashes is also incorrect . . . even in an ideal stroboscopic display with infinitesimally brief pulses, the temporal power of retinal stimulation during fixational drift is more broadly distributed at high than low spatial frequencies, in the same way that it occurs for natural stimuli.”

This is another occasion when Rucci et al. are concerned with the input to the visual system and not with how such input may be processed. They even go as far as falsely attributing to me the following: “Gur's intuitive assumption that the stimulus . . . cannot be reformatted by eye movements.” To clarify the issue, let's look at the stimuli in Kaung et al. (2012), where each pixel was flashed for 0.1 ms every 10 ms (a 100 Hz refresh rate). So, during each 100-ms drift, the image was flashed on the retina for a total of 1 ms. To generate a timecode, several neurons must cross each input element. When each pixel is flashed for 0.1 ms, there is no drift across the image, and no timecode can be had.

**At an empirical level, retinal stabilization results obtained with CRTs have been replicated in non** **strobe displays (** Li & Rucci, 2024 **)**.

Using nonstrobe displays is an improvement over using CRT ones, since during regular nonstabilized presentations, luminance does not vary. However, under image stabilization, to compensate for shifts in eye position, the image must be turned off and then turned on at a new position. There is also ∼10-ms delay in position updating (Lin, Intoy, Clark, Rucci, & Victor, 2023). Consequently, the image is not truly stabilized but is repeatedly flashed at several adjacent locations. Thus, similar to experiments using CRT monitors, “stabilized” images cannot provide any insight into processing by the drifting eye since the image interacts with the retina for durations that are too short for any significant drift.

It is ironic that in their attempt to show that flashes differ fundamentally from saccades, Rucci et al. argue that the excellent visibility of flashed images is not indicative of normal vision in that “flashed stimuli are powerful, but unnatural, transients.” But now we are told that there is no problem in using pulses to study the benefits of drift in normal vision.

In an attempt to play down the implications of using a 0.1-ms persistence/pixel display, Rucci et al. state, “It is also worth noting that CRT persistence is longer than what assumed by Gur, see Figure 3B in Elze (2010) or Figure 7 in Santini, Redner, Iovin and Rucci (2007), which would further contribute to spread power across temporal frequencies.” In the Perspective, I cited Elze (2010a), which shows that the CRT green phosphor single-pixel persistence was 0.1 ms. Rucci et al. blatantly ignore this study and refer to Elze (2010b), which shows a 1-ms red phosphor persistence for hundreds of pixels (10 lines; Figure 3B). Santini et al. measured the persistence of a fairly large square containing thousands of pixels.

### Concluding remarks

It has long been established that space is represented in V1 as an anisotropic 2D map where each cortical location contains neurons with feature-selective RFs, such that following saccade landing, image elements evoke responses in corresponding V1 cells. Thus, an image evokes a unique V1 response pattern that faithfully represents not just the relative spatial locations of image elements but also their spatial characteristics such as orientation or size. This description accommodates the well-known need for time-transients since the eye does not stay on any ROI for more than ∼250 ms.

Despite the many physiological and behavioral studies that are consistent with that view, it has been challenged as ignoring the dynamic nature of the visual system. While challenges are essential to scientific progress, they should be based on demonstrating fundamental flaws in the established view, solid empirical evidence, and an alternative model. As shown in this Letter, the STT encoding challenge does not fulfill any of these requirements. That the STT theory persisted for so many years despite such obvious shortcomings can be attributed to advocating the presumed benefits of reshaping spatial inputs by FEMs while shying away from dealing with neural mechanisms that are required for substantiating such inputs.

In my Perspective (Gur, 2024) and in this Letter, I pointed out that, unlike the STT theory, the pure spatial approach describes an efficient, consistent, and fast transmission and representation of the visual image. I have shown that the dynamic behavior of the visual system results from the phase-locked neural volleys initiated by the landing saccades. These volleys provide the necessary and sufficient information for the visual representation of the outside world. That the drifting eye does not generate significant information explains why vision is not blurred during drift.

## Supplementary Material

Supplement 1

Supplement 2
