# Supplementary figures and images for: Seeing on the fly: No need for space-to-time encoding; saccade-generated transients enable fast, parallel representation of space
Source: J Vis. 2025 Sep 4;25(11):4. doi: 10.1167/jov.25.11.4 (PMC12416515; doi:10.1167/jov.25.11.4)

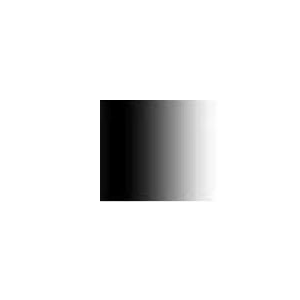

Supplement: Supplement 1 [file jovi-25-11-4_s001.gif]

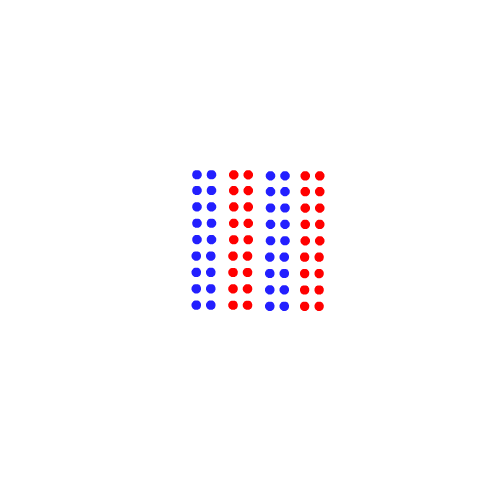

Supplement: Supplement 2 [file jovi-25-11-4_s002.gif]
